# Exploring the perception of key stakeholders toward khat policy approaches in Ethiopia: a qualitative study

**DOI:** 10.1186/s12954-023-00858-y

**Published:** 2023-08-26

**Authors:** Kassahun Habtamu, Solomon Teferra, Awoke Mihretu

**Affiliations:** 1 School of Psychology, College of Education and Behavioral Studies, Addis Ababa University, Addis Ababa, Ethiopia; 2 Department of Psychiatry, School of Medicine, College of Health Sciences, Addis Ababa University, P.O. Box 1176, Addis Ababa, Ethiopia

**Keywords:** Khat use, Khat use disorder, Khat harm reduction, Policy approaches, Perception, Qualitative study, Ethiopia

## Abstract

**Background:**

Khat is an amphetamine-like plant, produced and commonly chewed in Ethiopia by a large group of the population. Although significant multidimensional harms of khat use have been reported, currently, there are no policies or organized activities against khat use in Ethiopia. Therefore, the current study aimed to explore the perception of key stakeholders toward khat policy approaches for Ethiopia.

**Methods:**

A qualitative study was conducted using focus group discussion (FGD) and in-depth interview (IDI). Ten stakeholders participated in the IDIs, and another 15 individuals participated in the FGDs. Key stakeholders were appropriately mapped and purposively selected based on their experience related to khat use and khat harm reduction. Interviews and FGDs were led by the study authors and were audio-recorded. The audio-recorded data were transcribed verbatim and then translated into English language. The data were analyzed using thematic analysis approach.

**Results:**

Participants preferred prevention and law regulation measures for khat policy approaches for Ethiopia. Proposed prevention and law regulation measures included regulating the transportation of khat, limiting the minimum age to buy and sell khat, prohibiting khat use at some public places, banning khat advertising and promotion, and imposing excise tax. Individual level khat harm reduction strategies were also proposed to be useful. However, the participants asserted that total khat ban in Ethiopia is not likely to be feasible and acceptable.

**Conclusion:**

Prevention, treatment or care for individuals with problematic khat use, law regulation, and harm reduction were preferred approaches by stakeholders for khat policy in Ethiopia instead of total banning.

## Background

Khat (*Catha edulis*) is a shrub or tree with psychoactive properties such as cathinone, cathine, and norephedrine, which all have a similar chemical structure to amphetamine [1, 2]. Community-based studies showed that khat chewing is a routine practice in Ethiopia [3]; it is considered as the second most widely used psychoactive substance next to alcohol [4]. The national prevalence of khat use in Ethiopia is 15.3% [5]. In some regions where there is cultivation of khat, such as the Harari region, the prevalence reaches up to 50% [6]. A systematic review of studies conducted in Ethiopia found that the pooled prevalence of khat use among high school and university students is 16.7% [7]. In a separate analysis, the prevalence of khat use among high school students was found to be 14.61% and 17.56% among university students. Another systematic review study found that the prevalence of khat use among university students in Ethiopia is 23.2% [7, 8].

Several factors are found to be significantly associated with khat use. These include male gender [8, 9], age group 19–23 years [9], peer pressure [10, 11], family history of chewing khat, having friends who chew khat, urban residence, high income and having habit of drinking alcohol [5, 8]. People chew khat for several reasons, such as to improve their concentration and performance during prayer, work, and academic activities; to avoid unpleasant feelings; and to spend their extended spare time [8, 12].

Studies show that khat use results in multidimensional harms. The World Health Organization Expert Committee on Drug Dependence (ECDD) [13] reported that khat use has cardiovascular (tachycardia, palpitations, hypertension, arrhythmias, vasoconstriction, myocardial infarction, cerebral hemorrhage and pulmonary edema) and respiratory (tachypnoea and bronchitis) adverse effects. Gastrointestinal adverse effects of khat use include dry mouth, polydipsia, dental caries, periodontal disease, chronic gastritis, constipation, hemorrhoids, weight loss, duodenal ulcer, and upper gastro-intestinal malignancy. A small-scale study found significantly higher chronic illnesses, such as heart disease and stroke among khat chewers compared to non-khat chewers [13]. A systematic review of studies conducted in Ethiopia reported that chronic khat chewing is associated with increased systolic and diastolic blood pressure [14]. Khat chewing is also significantly associated with cardiovascular diseases [15]. There is a strong evidence related to the adverse effect of khat chewing on periodontal, oral health [16, 17]. A short-term prospective cohort study highlighted an increase in depressive symptoms as a result of khat use [18]. A study found that Khat use has adverse effects on short-term memory, but not on long-term memory [19]. A systematic review of studies showed that the association between khat use and psychosis, major depression, and bipolar disorder is inconsistent [20]. However, the impact of khat use on mental illness is likely to be significant [21]. Another commonly reported harm as a result of khat use is that it is a gateway for the use of other psychoactive substances, such as alcohol and cigarette [22–25]. A systematic review found that the prevalence of daily tobacco use among adults with Khat use is 17%, and this is higher among students (i.e., 30%) [26].

Social problems such as frequent quarrel among family members, breach of family ties, neglect of education, less care for children, encouraged prostitution, and addiction are linked with khat chewing [27, 28]. Spending money to maintain the habit of khat chewing and wasting working hours attending khat ceremonies lead to family neglect, domestic violence, and consequently to divorce [12, 29, 30]. Studies also reported such harms of khat chewing as loss of work habits, excessive expense, debts, and inadequate saving [30, 31].

There has been an increase in studies investigating the prevalence and adverse consequences of khat use; however, there is lack of research on the ways to reduce the harms of khat use. In Ethiopia, currently, there are no organized activities for discouraging khat production, sell and consumption. Although khat is a public revenue and is supporting the economy, there is no clear or assertive policy of support for production and sell. Some local level regulations and prohibition interests have been observed in the past from the Harari Region [32] and recently from the Amhara region. The government in Ethiopia has instituted some limits on domestic consumption, including bans on “khat house” venues [33]. But we observed that the enforcement is a campaign rather than a routine activity. In public universities and some workplaces, khat ban has been instituted [34, 35]. There were also regulations adopted by some regional states, such as Benishangul Gumuz, Gambella, and Tigiray Regions to control khat production, distribution and sell [36], but end up without being successful. The Ethiopian Federal Parliament passed a new excise tax regulation on khat in 2012, which aimed to reduce domestic consumption and to increase export [37]. These two aims of the excise tax regulation, however, look contradictory. Overall, there are no evidence-based and coordinated national level interventions to reduce harms associated with khat use.

To the best of our knowledge, there is dearth of evidence on the perception of stakeholders on the effectiveness of the different policy approaches (e.g., banning, law regulation, prevention and harm reduction) to reduce harms associated with khat use in the Ethiopian context. This study, therefore, aimed to explore the perception of key stakeholders toward khat policy approaches in Ethiopia.

## Methods

### Study design

A qualitative study was conducted using focus group discussion and in-depth interview. Qualitative study was done to explore the perception of key stakeholders toward the different policy approaches to reduce khat use harms in Ethiopia.

### Study site and context

The study was conducted in the different Federal Ministerial offices of Ethiopia and Addis Ababa City Administration. Key stakeholders from the Ministry of Justice, Ministry of Health, Ministry of Women and Social Affairs, Civil Society Organizations (CSOs) working on addiction and substance use, and healthcare providers (rehabilitation workers) from hospitals which have Addiction Rehabilitation Centre were interviewed. In-depth interviews and focus group discussions were also conducted with individuals who are previous service users or people who have recovered from khat use disorder.

From our previous experience, we have known that all of the above key stakeholders have interest to participate in activities related to khat policy options. They also have their own different types of initiatives in this regard. Thus, they accepted this study and were willing to participate. This study was also an opportunity to bring several stakeholders on board and work on khat harm reduction.

### Participants and sampling

The study was conducted in Addis Ababa, and participants were selected from federal level ministerial offices, Civic Society Organizations (CSOs), hospitals which have Addiction Rehabilitation Centre and individuals with experience of khat use. Specifically, the key stakeholders who participated in the study include experts or key informants from the Ministry of Justice, Ministry of Women and Social Affairs, Ministry of Health and health facilities which have addiction treatment center. Our assumption is that these experts are directly or indirectly involved in awareness creation activities, regulation and treatment of khat use. We also included individuals with experience of khat use and key informants from CSOs working on khat use. In addition, a community leader and a religious leader were interviewed as they are opinion leaders and have better access to information in the community related to khat use, production, distribution and sell. Actual sample size for the in-depth interview (IDI) and focus group discussion (FGD) was determined up on theoretical saturation. Ten participants were interviewed individually and the rest 15 involved in the two FGDs. We purposively selected experts or key informants from key stakeholder organizations. Participants were selected based on their previous work experience related to khat use or other psychoactive substance use.

### Methods of data collection and procedures

Data were collected from key stakeholders and people who use khat or had used khat using in-depth interviews (IDI). We were not able to use FGD for key stakeholders as it was difficult to bring them together from different offices. The experiences and power hierarchies of these participants are also different, and running a comfortable discussion was difficult. So, FGD was conducted with khat users or who had used khat. FGD was used with people who use khat or had used khat to elicit more information through their interaction and to encourage sharing of their experiences [38]. In each FGD, seven or eight respondents were involved.

Both the IDIs and FGDs were conducted face-to-face in Amharic language, the official language of Ethiopia. All FGDs and in-depth interviews were conducted by the study authors (who are faculty members at Addis Ababa University). Both of these researchers have training and several years of experience in qualitative data collection. While these researchers were moderating FGDs, their respective note takers or field assistants summarized the discussions and noted the non-verbal communications. In-depth interviews with key stakeholders were conducted in their respective offices, whereas the interviews with the community representative and religious leader were carried out in a comfortable place in their residence areas including their home and church/mosque. The FGDs with khat users were carried out in a comfortable place in their residence areas. Privacy and confidentiality of participants of the interviews and FGDs were assured at all times. All interviews and FGDs were tape-recorded, with the consent of the participants. Generally, in-depth interviews lasted between 40 and 80 min, whereas FGDs lasted between 60 to 120 min.

We developed and used a semi-structured topic guide for conducing the IDIs and FGDs. The topic guide was developed iteratively as data collection and preliminary analysis continued [38]. The content of the topic guide focused on the perception of stakeholders about harms associated with khat use and policy interventions to khat use harm reduction, actions required to reduce harms associated with khat use and existing initiatives for khat harm reduction.

### Data analysis

The audio-recorded data were transcribed verbatim by experienced transcribers and then translated into English. The data were analyzed using thematic analysis approach [39]. After reading the transcripts and listening to the audio data, coding was done by one of the authors in consultation with the other author. Higher-order codes were derived from the primary codes with thorough discussion between the first and second authors. Similarly, overarching themes were developed from the higher-order codes. Illustrative quotes were selected for each theme and subtheme. After the qualitative data are organized around the identified themes, emerging similarities and differences in responses were recorded under each theme. The results were then presented using descriptions that reflect the mix of responses.

### Data quality control and assurance

Both the IDIs and FGDs were conducted by the study authors who have experience in qualitative data collection. The study authors did the IDs and FGDs ensuring that they respect the participants and acting in a professional manner and that the interviews maintain the privacy of the individuals. All the IDIs and FGDs were audio-recorded. The audio data were then transcribed into the local language and then translated into English before coding was started. All the data, including audio-recordings and transcripts, were stored in a secured place using password-protected audio-recorder, computer and hard driver.

### Ethical considerations

The study protocol was reviewed and approved by the Ethics Committee of the School of Psychology, College of Education and Behavioral Studies, Addis Ababa University (Ref No.: AAU/CEBS/SoP/Staff/01/22). The study was conducted being adhered to all the relevant national and international legislations and guidelines relating to the conduct of clinical and healthcare research. We anticipated that the risk of any harm is minimal since the key stakeholders respond with their experience and professional capacity. Participants were invited to involve in the study after making sure that they are well informed about the study. We sought written informed consent from each of the participants. The participant information sheet and the informed consent form were written in a clear language, enabling participants to understand the aim, procedures and implications of the study, and the nature of participation.

Participants were given the electronic copy of the information sheet to keep for their reference. Participants were informed of their right to withdraw from the study at any time without having to give a reason and without having any adverse consequences. Participants were also informed as to the specific final withdrawal date in the information sheet. Confidentiality and anonymity of data were ensured throughout the research process. Data and participant related files were stored in a password-protected computer and hard disk. Data collectors were trained on COVID-19 and related safety. All the necessary precautions to prevent the transmission of COVID-19 were implemented during data collection.

## Findings

All of the participants were male, except one. Participants were from diverse age group (ranging from 27 to 68 years), educational level and employment status (Table 1). All of the khat users were males. The mean age of participants who used to chew khat was 33.6 years (SD =  ± 7.0). About half of the participants were khat users (n = 15); nine were mental health professionals, experts from the Ministry of Health, Ethiopian Food and Drug Administration, CSOs, Ministry of Justice and Ministry of Women and Social Affairs. One of the participants was a religious leader.

**Table 1:** Characteristics of the participants

Characteristics	Number	Participant ID
*Age*		
25–35	6	
36–45	7	
46–55	9	
56 and above	3	
*Gender*		
Male	24	
Female	1	
*Education*		
Secondary education	11	
Undergraduate level	8	
Postgraduate level	6	
*Employment status*		
Self-employed	9	
Unemployed	2	
Employed	14	
*Participant type*		
Khat users	15	FGD 01 and FGD 02
Ministry of Health	1	IDI131
Ethiopian Food and Drug Administration	1	IDI132
Civic Society Organization	3	IDI33, IDI134, IDI135
Religious Leader	1	IDI136
Mental health professional	2	IDI37, IDI 138
Ministry of Women and Social Affairs	1	IDI139
Ministry of Justice	1	IDI140

Participants suggested different types of policy approaches: prevention targeting individuals, families and communities; law regulation; banning; care or treatment for problematic khat users and other harm reduction strategies.

## Khat use prevention

Almost all of the participants suggested prevention activities (e.g., education and awareness creation activities) as important measures to prevent and reduce harms of khat use. Diverse platforms for public awareness were suggested including stage meetings (face-to-face meetings), using posters, brochures, flyers, billboard, using main stream media (TV and radio), websites and social media. Efforts to create awareness among the public using such platforms are minimal in Ethiopia except small-scale face-to-face meetings. Participants were critical of the media, particularly the main stream media, in that they are not working to raise the awareness level of the public about khat use, while there are many publicly reported societal problems associated with it.

A few participants indicated that existing government plans and programs focus on education about khat use behavior. A key stakeholder from the Ministry of Women and Social Affairs stated the following:

Our office is working on awareness raising targeting prevention and harm reduction of khat use. Since there are national economic benefits from khat, we found that banning khat use would not be acceptable and feasible. [IDI 139]

Although there were no formal advertisement and promotion for khat use in the mainstream media, khat shops did their own advertisement such as using posters and social media. It is also common to see actors chewing khat in local movies which is not the case for alcohol. Participants called action against such kinds of vicarious learning. Khat use by law makers and celebrities at public places was also mentioned as reinforcing for young people. In this regard, one of the FGD participants highlighted:

When you see lawyers and policy makers buy and chew khat, you will think that it is meaningless to struggle for law regulation since the issue is not taken seriously by decision makers. Above all, this would reinforce others who model them. What would be the lesson for a student who observes his or her teacher chewing khat? In the past, such groups of the society used to buy and chew khat secretly but now they make it public. [FGD 02]

Khat use prevention is reported to be a best solution to protect individuals from the potential harms of khat use.

I highly believe in the power of abstinence more than other policy options because in the case of substance use disorders there is impairment of control. Once you start chewing, it is hard to control the chewing both in terms of time and amount. This implies harm reduction does not work. [IDI 138]

As a religious leader, our holy Bible prohibits the use of khat which distorts the mind. I think this also works for holy Quran. So, we are teaching and will teach about abstaining from use of khat. We still want to remind the government to expand youth recreational centers where they can spend their spare time. [IDI 136]

Some of the participants favored awareness-raising programs on the harms of khat use, and therefore khat users will have informed decision on their use. On the other side, some argued that it would be impossible to curb the current problematic trend of khat use without law regulation. They also highlighted that it would not be possible to prevent minors and other hazardous khat use pattern with the current status of the country on khat.

Our actions should be person-centered care as this would bring an impact. So, I don’t believe in law rather I believe in human -centered approaches. But the law regulation would save children. They should be protected from the evils of the world. [IDI 134]

## Law regulation

Since khat use is embedded within the sociocultural context in many parts of Ethiopia, participants suggested law regulation against khat and khat use as a plausible and feasible policy option. The macro- and micro-economic attractions of khat were also reported as additional reasons for law regulation rather than banning.

I don’t buy the idea of totally banning khat since there are macroeconomic and micro economic benefits of khat; both for the government and farmers. But there should be regulation about planting, transporting and selling. [IDI 133]

All groups of participants, including stakeholders from the government sector (health, social and justice sector), were interested for law regulation against khat. They asserted that both khat users and khat producers are not getting benefits with the current status of khat except brokers. Appropriate law regulation measures could benefit everyone in the chain of the khat market. This could happen even without reducing the current amount of revenue being collected from khat.

When we conduct meetings about the implementation of our strategies for alcohol and tobacco control, the community frequently questions the issue for khat. They urge a law regulation for it. [IDI 132]

Responses against khat use needed to be grounded on the multidimensional harms of khat use and khat use disorder. Although the harms of khat use are not well communicated to the public, there are sufficient evidence in some areas such as the addictive nature of khat or the validity of khat use disorder and its socioeconomic harms. In few areas such as physical health harms, participants call for additional strong scientific studies.

In-depth interviews with key stakeholders showed that there are dialogue and debate about khat use among few interest groups such as think-thank groups, civil society organizations and experts from the health sector. However, there is no particularly assigned and responsible body from the government to move forward the response against khat. The lesson from tobacco and alcohol law regulations showed that the Ethiopian Food and Drug Administration (EFDA), under the Ministry of Health, took the responsibility of the coordination of activities and other responsible bodies throughout the process including Ministry of Justice, Council of Ministers, and the Parliament. In the case of khat, however, EFDA is not officially assigned to lead the activities related to khat law regulation. A participant from EFDA reported as:

I have been involved for tobacco and alcohol law regulation. The laws we set are well recognized ones, especially laws related to tobacco. Thus, Ethiopia has been awarded five times for this. We managed to discharge our responsibilities successfully. We can also do similarly with khat if the assignment is given to us. [IDI 132]

The reported law regulation measures for khat use included regulating the transportation of khat, limiting the minimum age to buy and sell khat, and prohibiting khat use at some public places and khat advertising and promotion.

At the top management level, there is an agreement on the regulation of khat but no one has taken over the responsibility. Different sectors such as agriculture, health, and education try to do their job separately rather than in coordination. There seems to be an interest to regulate the transportation of khat, and setting age limits to market and consume khat. [IDI 140]

Another suggested measure by participants to reduce the demand for khat was imposing excise tax. Participants reported that excise tax would increase the price of khat and this would be one of the effective mechanisms to reduce the demand for khat.

Now, khat is getting expensive. It has become something that you do not want to use on most days. It is really hard to afford the price of khat to use it on a daily basis and imagine what will be the decision of the khat user if the cost increases more than the current price. Therefore, I recommend the government to use this as an effective solution to decrease the demand for khat and to reduce the harm. [FGD 01]

Availability of and easy access to khat was mentioned as one factor for khat use. Thus, participants suggested law regulation targeting the transportation and sell of khat and setting an age limit to buy and sell khat.

### Regulation targeting the transportation of khat

Some participants saw khat at public transport for the first time, and this is considered as an opportunity to be curious about it. Participants endorsed that transporting khat should be with cargo not by public transport. They asserted that transporting people and khat together would create familiarity and attraction to the khat.

How could a drug [khat] and human being travel together in a bus or mini-bus? This is weird. There are also many people who chew in the bus. They even ask you if you chew. Thus, transporting khat with public transport should be prohibited. [FGD 01]

### Regulating the sell of khat

Almost all of the participants endorsed banning khat use at some public places especially in and around schools and health care settings. For instance, one of the in-depth interview participants had the following to say:

There are some attempts to regulate khat use around school areas. Although it is not strict, the Ethiopian government doesn’t offer trade licenses for khat shops around school areas. The Ministry of Education, is trying to follow-up this. [IDI 140]

Another in-depth interview participant said*:*

We see khat outlets in front of schools and students hide themselves there skipping classes or after class. To shape the character of students and support them for holistic development, it is expected from us to minimize environmental risk factors for khat use. Thus, we need to strive for the realization of a policy framework from low level to high level. [FGD 02]

### Early age limit

Most of the participants reiterated that unless age limit for buying, selling and chewing khat is set, it would not be possible to protect children from khat use since exposure could be a reason to initiate khat use at an early age. Currently, a significant number of children are taking part in khat farms and markets. They work at their family khat farms or others for paid work. In cities, children also work as messengers of khat from khat shops to khat consumers. Thus, although it is possible to safeguard children from such kinds of engagements using the existing national laws and policy frameworks, it is still recommended to limit the early age to buy, sell and chew khat.

Starting khat use at early age increases the chances of developing khat addiction and experiencing many khat related and khat specific harms later in their life. Thus, we need to protect children from pushing factors to khat use including exposure. [IDI 138]

### Establishing alternative livelihood

Almost all of the participants suggested alternative livelihoods for people whose life is dependent on khat. Participants also suggested that the government and other concerned stakeholders should discourage khat production as a livelihood. Participants emphasized that any policy against khat use could leave people who are at khat market chain, ranging from farmers to retailers, at economic risk. In the opposite of this, we found a frequent claim by participants for law and order about khat production, both wholesales and retails, and consumption in the country to prevent adverse impacts on society. Participants mentioned the status of alcohol and tobacco law regulation as example. They justified that even though the country has set and implemented strict law regulation for alcohol and tobacco, producers and traders are still profitable. Not only individual level livelihood but also national level alternative income should be planned since the international market of khat looks unreliable. Different countries are banning khat, and this is negatively affecting the national export of khat.

In Ethiopia, the issue of khat is not in orderliness. It is neither controlled nor banned. While khat consumption and production are legal, opening khat café for business is illegal though the enforcement is weak. I do not think this law is good and would be efficient. [IDI 133]

We should be concerned if our khat export destination countries ban khat entering their country. The government needs to plan ahead. Considering khat as a sustainable source for foreign currency is not appropriate. We have to make our economy independent from khat. [IDI 135]

## Total khat ban

Most of the participants believed that banning the production and use of khat in Ethiopia currently seems unacceptable and unrealistic due to several reasons. Participants highlighted that the economic benefits, the cultural basis of khat in many parts of Ethiopia, lack of preparedness, the political unrest in the country and having other national priority agenda would hinder Ethiopia to move for banning khat. Participants anticipated the expansion of khat contraband and crime if the country bans khat before creating public awareness and trying other policy options. There were other few participants, however, who argued banning as an appropriate policy option. They argued that banning khat partially or mere law regulation would create confusion for the public. When laws are not uniform there would also be implementation gaps. Law enforcement bodies could also compromise the proper law enforcement for different reasons. They stated that if the production, sells and consumption of khat are prohibited for everyone in every circumstances, it would be possible to immune everyone from the harms of khat use. Participants understood this kind of law as clear and its enforcement is also expected to be effective.

I believe in the power of law that prohibits khat use. For example, we know driving without wearing a seat belt is risky, but, in the past, many people used to drive without wearing seat belt before the law was introduced. After the law, now, everybody wears seat belt during driving due to fear of the punishment. This law is clear and uniform. I suggest something similar for khat. [FGD 02]

## Khat harm reduction

We found no evidence on the successful application of any khat harm reduction approach neither at individual nor at the community level. Some participants reported lack of information about the design and delivery of harm reduction interventions for khat use. Khat harm reduction strategies reported by the participants are any efforts to minimize harms associated with khat use. These included keeping the safety of the khat itself, chewing in a safe environment, limiting the amount and frequency of khat use, avoiding the use of other psychoactive substances and adhering to a healthy lifestyle which reduces vulnerability of the health harms of khat. In order to keep the safety of khat, participants suggested avoiding pesticide and washing the khat before use. Participants also suggested chewing khat in a well-ventilated space to prevent infectious diseases such as COVID-19 and tuberculosis. Khat cafés or houses used for khat chewing and shisha smoking, which are mainly preferred by young people, are poorly ventilated since they should be out of the reach of the police. It was also highlighted that under-age boys and girls work in these houses, who might also engage in risky sexual behaviors. Thus, closing such kinds of khat outlets would also prevent risky sexual behaviors.

Regarding the physical limit of khat, some of the participants argued that there is no any safe limit for khat. There are people who engage in risky behaviors such as smoking tobacco and drinking alcohol after they chew even a small amount of khat. The physical limit of khat in amount, length of khat session and frequency of khat use was found effective and feasible for some individuals, although some others found themselves incapable due to impairment of control over the use.

Although, I know individuals who come to health facilities because they could not chew by controlling the amount of khat they use, there are also individuals who only chew at weekends or in a small amount. [FGD 01]

For people at the problematic stage of khat use, I recommend quitting after treating the symptoms related to withdrawals and working on relapse prevention. I don’t think harm reduction would be effective for these groups of people. Of course, if someone is good at diet, reduces the amount of khat he or she chews, engage in pleasurable activities, it could be possible to prevent some additional harms of khat use. [IDI 137]

### Risk factors for harms of khat use

Most of the participants agreed that younger people are at greater risk to khat use harm due to impairment to control their khat use as there is peer pressure and brain adaptation to the effects of the khat. The impairment to control over the khat use could further result in socioeconomic harms among young people. People who chew khat for functioning over the night such as students, truck drivers and commercial sex workers were also found to be more vulnerable to the harms associated with khat use. Younger people also usually chew khat for recreational purpose more than for cultural reasons. In areas where khat use is part of the cultural norm, people were likely to start chewing at an early age and could be affected by the khat more than others. Therefore, the probability of continuously chewing khat and being at risk of problematic khat use among younger people was higher. A participant highlighted why young people are at risk of engaging in khat use as follows:

Minors would take emotionally informed decisions including khat use since their frontal lobes do not fully develop by their age. They also want to experiment khat use when they are invited by their peers since they want to get social approval. Thus, there should be a policy that safeguards them. [IDI 137]

Many of the participants indicated that people who chew khat before meals were at risk for experiencing adverse consequences from khat use. Individuals who chew khat as a coping mechanism for their stress and distress were also vulnerable to experiencing problematic khat use. Khat users believed khat enhances feeling high or pleasure and temporarily helps to manage difficult emotions. A participant in one of the FGDs argued:

Khat seriously harms your body when you often chew without eating food. I always eat well if I need to chew khat. Unless you eat before, khat suppresses your appetite after chewing. People who often don’t eat well either due to lack of appetite or because they buy khat on the cost of buying for their food have a low body weight and, thus vulnerable for physical health harms due to nutritional deficiency. [FGD 01]

I later realize that if you often chew khat as coping from distress, you will later develop khat addiction and serious harms due to chronic use. The unfortunate is the khat use will also induce additional distress after some time. It is also very hard to look for other problem-solving strategies, once your mind is conditioned for khat. [FGD 01]

### Measures to reduce khat related harms

We found community-based activities targeting individuals, families, schools, community associations and the community at large as recommended measures against khat use. In schools, establishing Anti Khat Clubs and educating young people to abstain from khat use were suggested as feasible and effective measures. Engaging religious and community leaders at all types of action against khat use including prevention and law regulation was reported as important and potentially effective. Religious and community leaders were also considered important to change practices in the community that reinforce khat use.

Education about khat use would be more effective when it’s delivered by religious and community leaders more than clinicians because there is better acceptance and trust. Clinicians are trained in a Western model and sometimes there is miscommunication and inability to understand the explanatory models of the local community. [IDI 138]

Key stakeholders from government offices and CSOs reported that there were efforts to engage the community for ongoing awareness raising activities about khat use and its associated harms. For instance, a task force which constituted different community members was established by the Ministry of Women and Social Affairs to raise awareness about the harms of problematic substance use but went without continuously being functional. Participants highlighted that community-based approaches to reducing harms associated with khat use are more effective than other approaches as they address the community directly.

We know that the community has some level of awareness about the harms of khat but there are minimal efforts to the actions against khat use, may be due to lack of coordination and mobilization. So, I recommend that professional associations, youth forums, youth representatives and religious leaders coordinate and mobilise the community to take action against khat use. [IDI 139]

Different strategies could be used to coordinate the different stakeholders and mobilize the community. Some of the participants suggested that the government should take the responsibility to coordinate the stakeholders and use its existing structure. Others suggested that Civil Society Organizations should take the lead for the coordination of stakeholders and mobilization of the community.

We [civil society organizations] don’t have the mandate to take over the ministry offices duty but we took the role of bringing key stakeholders on board to work against khat use. Thus, we established lobby groups which include practitioners, think tank groups such as Forum for Social Studies and Ethiopian Economic Association. [IDI 133]

Some of the participants emphasized that recommended and evidence-based strategies should be used to raise public awareness and to mobilize the community.

We use participatory video and drama. These are very important tools to initiate dialogue and motivate the community for action. We learned that there are interests from the community but there are no platforms which translate their interest and participation meaningfully. There should be very good coordination and communication. [IDI 134]

Once the community is well aware and all the concerned bodies come on board, a lobby group can be established. Almost all of the participants suggested that all actions against khat and khat use needed to be multi-sectoral and inter-regional. All the actors who are involved from khat production to consumption should come on board and play a significant role to prevent khat use or reduce the harms of khat use. Participants who have experience in policy making suggested that any law regulation or banning should be done with inter-regional collaboration; otherwise, there would be differences in commitment to enforce any laws related to khat and khat use.

If one region prohibits khat use, people can easily get it or import it from the neighboring region. They will also not be convinced by the law if it is not national. [IDI 135]

It is resource demanding to prevent khat use or enforce laws against khat use unless resources are mobilized from concerned sector offices or key stakeholders. I also observed different level of motivation and task shifting for actions against khat use. Thus, with this level of motivation and commitment, any law from the law-making body will not be effectively implemented and will not be strongly supported by the key stakeholders. [IDI 140]

## Treatment for people with problematic khat use

Participants who have clinical experience reported that there were individuals who seek help with problematic khat use. A few of these individuals were admitted to addiction rehabilitation centers. However, there were no treatment guidelines, assessment tools and tailored interventions specific to khat use disorder.

On the streets, I witness a lot of individuals who chew khat or hold the leftovers of khat who also manifest behaviors similar to individuals with mental health conditions. Then, I ask to myself there should be a link between khat use and mental illness so that there should be a care to assist them to recover from both of their conditions and key stakeholders should be responsive to this. [IDI 131]

Respondents who have clinical experience highlighted that khat use disorder rehabilitation like other substance use disorders should be comprehensive targeting health, life skills, social and vocational needs of the individuals. Respondents accepted the limitations of rehabilitation centers and suggested a type of rehabilitation adapted for khat use disorder which should not entirely be institution-based.

Establishing a rehabilitation centre is expensive and demands resources such as trained personnel. Rehabilitation centres do not reflect the real-life condition of people. People usually drift from their social capital and skills when they stay there for long. Thus, we should not take institutional rehabilitation centres as a gold standard model of care. [IDI 137]

Since the withdrawal symptoms of khat use disorder are not life threatening and may not warrant detoxification, psychosocial focused rehabilitation would likely to be effective. These included life skills training and social support that assist their disengagement with khat and recovery from khat use disorder. In this regard, a participant who was a khat user stated:

It is not too difficult to be free from khat use disorder. As evidence, my friends and I who used to chew khat daily don’t chew during fasting season (i.e. remedan). Then we resume chewing khat because we are unemployed. Thus, I believe that if I had serious engagements such as work, I would stop chewing khat. Therefore, some level of motivational enhancement and availing job opportunities would help problematic khat users to recover. [FGD 02]

## Discussion

In this exploratory qualitative study, we investigated the perceptions of several stakeholders on the possible actions against khat and khat use in the Ethiopian context. The need for any actions against khat and khat use emanates from the multidimensional harms of khat and the prevalence of khat use in the general population. A significant proportion of people in Ethiopia chew khat on a daily basis [6]. There are also enormous socioeconomic adverse effects associated with khat use such as frequent quarrel among family members, wasting working hours, more debits or inadequate saving, domestic violence, breach of family ties, neglect of education, and less care for children [12, 27–30]. Another frequently reported harm as a result of Khat use is that it is a gateway for the use of other psychoactive substances such as alcohol and cigarette [22–25]. Although strong evidence is warranted in few areas such as health adverse effects of khat, the impact of khat use on periodontal, oral health has been consistently reported [16, 17]. Chronic khat chewing is also associated with increased systolic and diastolic blood pressure [14] and cardiovascular diseases [15]. Khat use disorder is a valid disorder which has to be recognized [40, 41].

We found prevention to khat use, khat regulation, total khat and khat use ban, and khat harm reduction interventions to curve the harms of khat use as possible approaches to khat and khat use policy in the Ethiopian context. Prevention of khat use, including education and awareness creation activities using different platforms, is important to prevent and reduce harms associated with khat use. We also found law regulation, targeting the transportation and sell of khat and setting an age limit to buy and sell khat, as an important action against harms of khat use. Imposing excise tax and prohibiting khat use at some public places were also found to be other options to reduce the demand for khat use.

While different policy options are suggested and implemented for different substances, discussion about policy options for khat use has been rare in Ethiopia. In many Western countries, however, the adverse consequences of khat use, mainly among migrant communities, had pressured policy makers and health professionals to ban khat use [42].

Although the status of khat is a controlled substance outside of East Africa and the Arabian Peninsula, we found that banning as a policy option in Ethiopia is less likely to be feasible and effective in the current condition of the country. Since Ethiopia is in an instable condition, banning khat will not be possible to enforce, and rather might be counterproductive. Moreover, the deep-rooted cultural basis of khat use in many communities of Ethiopia and the economic attractions of khat for the Ethiopian macro-economy could make banning khat impossible in a short period of time. In many parts of Ethiopia, people chew khat during social gatherings like weddings and funerals and to host guests as well as to meet other people and exchange of ideas [43].

High levels of unemployment in Ethiopia, particularly in urban areas, and the pursuit of pleasure may also contribute to the reluctance to consider a legal ban on khat. However, the underlying factors driving the continued popularity of khat, despite known harmful effects, require further exploration. The negative perception of khat use is often attributed to its combination with other psychoactive substances, including alcohol and/or cannabis, in both Ethiopia and other contexts, such as Uganda [44]. Furthermore, there is a notable absence of recommendations from various national agencies including the United Nations Office on Drugs and Crime and the World Health Organization [13].

Banning, as a policy option, has also a number of limitations as reported from other countries for different drugs. Banning could lead to massive arrests and incarcerations. Marginalizing problematic substance users (khat users in our case) who need health care, expansion of illegal trade, corruption among law enforcement and other bodies are some of the limitations associated with banning [45]. The largest production of khat in Ethiopia is limited in some areas and population groups. Therefore, banning and law enforcement has to target these areas and subgroups, and this is likely to be interpreted as a partial action and would exacerbate the existing ethnic tension. Lower capacity of the nation to avail alternative livelihood for people engaged in the chain of khat market could also push people to illegal khat trade to fulfill their basic needs. In this regard, Klein stated that the consequence of war on drug policy is reported to be more devastating than the drug use itself [46]. In different settings, the significant economic attractions of khat for khat producers, khat sellers (retail and wholesale) and generating foreign exchange also requires evidence-based khat regulation than total banning [47].

The current study asserted Khat prevention programs and law regulation as potentially effective and feasible policy options. Some previous studies showed that substance use prevention approaches applied to particular youth contexts reduce frequency as well as amount of use [48]. However, prevention programs have been critiqued for their narrow focus on preventing or avoiding use, as opposed to equipping the users with skills in identifying and mitigating harms that may occur within the context of choosing to use [49].

Although a significant number of young people use khat, it is also considered unacceptable behavior by religious leaders and elderly people in Ethiopia. This could open a door to create social contexts where khat use is not reinforced and play an important role to prevent khat use among young people. But khat information should be presented to young people in a manner that resonate them. The reasons and social context of young people with khat use should be acknowledged and addressed [50]. The current study identified strategies to engage the whole community such as the use of participatory videos in order to understand the context of khat users. Awareness raising activities through anti-khat clubs in schools and universities could also be more acceptable for young people and information from their peers could also be considered more trustworthy rather than education programs led by adult authority figures [51, 52].

The current study showed that law regulation targeting the transportation, distribution and sell of khat and setting age limit to buy and sell khat is an important policy approach. The study further indicated that demand for khat can be reduced by imposing excise tax, prohibiting khat use at some public places and strictly prohibiting khat advertisement and promotion. These strategies are in line with the international approaches for actions against alcohol and tobacco use [53, 54]. Strategies against problematic substance use are also suggested to prevent non-communicable diseases in addition to preventing the respective substance specific harms. For the case of tobacco and alcohol, different countries, including Ethiopia, mobilized resources and are able to implement their respective policies [55].

Unless the actions against khat and khat use are multi-sectoral and inter-regional, it is not likely to be effective to set and enforce laws and regulations. There were previous unsuccessful efforts in different regions in Ethiopia such as Harari et al. [32, 36]. Thus, coordinated national level and multi-sectoral actions should be designed and enforced.

There is an absence of evidence or evidence of absence for any strong khat harm reduction strategy from the perspective of khat users or key stakeholders as well as from previous studies on other substances, such as injectable drugs [56]. Although there is no established khat harm reduction strategy, some of the harm reduction strategies suggested to be useful in this study could work for chronic khat users who must continue chewing khat for different reasons. We found such harm reduction strategies as keeping the safety of the khat itself, chewing in a safe environment, limiting the amount and frequency of khat use, avoiding the use of other psychoactive substances and adhering to healthy lifestyles to be important with specific to khat use. Therefore, even though law regulation was highlighted as very important policy option, these harm reduction strategies can be concurrently implemented. Some of the health harms of khat use are likely to appear after many years of chewing khat and this might reduce any types of efforts including establishing and implementing harm reduction strategies.

Considering the controversies about the concept and definition of ‘harm reduction’, we suggest the ‘broad’ definition rather than the ‘narrow’ for the context of khat [57]. For the broad definition, it indicates any intervention that reduces a known harm, including approaches to reduce risk and vulnerability. This allows to considering khat initiation at early age and chewing before having meals.

The main implication of the current study is for policy makers to consider the different policy options for khat, but focus on those which are likely to be feasible and effective. We asserted that there should not be a dichotomous understanding or just considering one of the policy options such as prevention or law regulation. We recommend that khat policy should encompass several of the different feasible and acceptable options, such as prevention, treatment or care for individuals with problematic khat use and law regulation.

One of the major strengths of the current study is the appropriate mapping of key stakeholder organizations and selection of key informants or experts from these organizations. The study is an important formative phase for future khat policy studies since there is no previous khat policy study in Ethiopia. The inherent limitations of qualitative research, such as lack of generalizability to the whole Ethiopia, should be considered and the consumption of the findings of the current study should be within the capacity of qualitative studies. In the current study, certain participants, specifically stakeholders, were from national or ministerial-level administrative units in Ethiopia. This allows for a degree of inference beyond just the city of Addis Ababa. However, it's worth noting that women remain challenging to engage as participants in the study. Therefore, we acknowledge that the opinions of women are underrepresented.

## Conclusions

Among the different policy options, khat regulation, at different levels and groups, is perceived to be the best response for problems associated with khat use in Ethiopia, but banning is likely to be unacceptable and infeasible at the current condition of the country. Law regulation targeting the transportation and sell of khat and setting an age limit to buy and sell khat are highly suggested. Demand for khat could also be reduced by imposing excise tax, banning khat use at some public places, creating public awareness, public education about the harms of khat, interventions for individuals with problematic khat use, and ban on khat advertising and promotion. In some situations, there are harm reduction options for problematic khat use. Bringing all of the key stakeholders on board and creating multi-sectoral collaboration are highlighted.

## Data Availability

Data used for this analysis will become available through the corresponding author at any time from now up on reasonable request.

## Abbreviations

CSO: Civic Society Organizations
EFDA: Ethiopian Food and Drug Administration
FGD: Focus group discussion
IDI: In-depth interview
ECDD: World Health Organization’s Expert Committee on Drug Dependence
